# Intraoperative Indocyanine Green Fluorescence Enables Primary Tumor Localization and Treatment De‐Escalation in SCCUP: A Case Report

**DOI:** 10.1002/hed.70240

**Published:** 2026-03-26

**Authors:** Nikhil Bellamkonda, Nitish Khurana, Eric Babajanian, Hamidreza Ghandehari, Jeremiah A. Alt, Richard B. Cannon

**Affiliations:** ^1^ Department of Otolaryngology—Head and Neck Surgery University of Utah School of Medicine Salt Lake City Utah USA; ^2^ Department of Molecular Pharmaceutics University of Utah Salt Lake City Utah USA; ^3^ Utah Center for Nanomedicine University of Utah Salt Lake City Utah USA; ^4^ Department of Otolaryngology/Head and Neck Surgery Mayo Clinic Rochester Minnesota USA; ^5^ Department of Biomedical Engineering University of Utah Salt Lake City Utah USA

**Keywords:** indocyanine green, SCCUP, transoral robotic surgery

## Abstract

**Background:**

Unknown primary cancer in the head and neck presents a difficult surgical treatment dilemma. Patients with squamous cell carcinoma of unknown primary (SCCUP) typically present with an enlarging neck mass found on biopsy but with no indication of primary site on diagnostic exams such as flexible laryngoscopy, CT, MRI, and/or PET/CT. Failure to identify primary sites eliminates surgical treatment as an option, pushing patients toward definitive chemoradiation with associated side effects.

**Methods:**

Indocyanine green (ICG) has been used to identify primary carcinoma in known oropharyngeal squamous cell carcinoma while using transoral robotic surgery (TORS). In this case, we injected ICG intraoperatively in a patient with SCCUP to help with the real‐time localization of ICG in the tumor.

**Results:**

ICG fluorescence successfully identified a previously undetected primary lesion within the oropharynx during TORS, which enabled precise surgical excision of the tumor.

**Conclusions:**

This case demonstrates the potential of ICG‐guided TORS to localize primary tumors in SCCUP patients, offering a pathway to surgical treatment and potentially reducing reliance on chemoradiation.

## 1 Introduction

Unknown primary cancer in the head and neck presents a difficult surgical treatment dilemma. An unknown primary cancer is defined as a malignancy, usually squamous cell carcinoma (SCCa), found within a lymph node in the neck without an obvious primary site [1, 2]. In the United States, the incidence of SCCa of unknown primary (SCCUP) has increased alongside rising rates of human papillomavirus (HPV) infection and related oropharyngeal cancers [3, 4]. Oropharyngeal cancer is now among the most common HPV‐related cancers in the United States, accounting for 80% of HPV‐related cancers in men, with an increasing incidence in the general population of 1%–4% per year for the past two decades [5]. The costs of caring for oropharyngeal cancer are high, with recent studies estimating between $52 400 and $146 100 per patient treated [6].

Patients with SCCUP typically present with an enlarging neck mass found to be SCCa on biopsy, but with no obvious primary site on either physical or endoscopic exam. Computed tomography (CT) and/or magnetic resonance imaging (MRI) in these cases have a primary site detection rate of 33%–50% [7, 8]. ^18^F‐fluorodeoxyglucose‐positron emission tomography (PET)/CT similarly has highly variable rates of detection, from 17% to 55.2% [9, 10]. Transoral robotic surgery (TORS) facilitates an option of enhanced visualization of the oropharynx; this has reported primary localization rates as high as 80% [11, 12].

Patients with SCCUP, especially those with large volume disease, typically undergo definitive chemoradiation with a larger radiotherapy field and/or chemotherapy [13]. This has been shown to be associated with increased adverse side effects [14]. Surgery can offer 91%–97% 2‐year progression‐free survival depending on the stage and adverse features identified and can be accomplished successfully after a non‐oncologic tonsillectomy [15]. TORS, however, requires identification of the primary site [16, 17]. Typically, for definitive chemoradiation of unknown primary head and neck cancers, the entire pharynx is treated with external beam radiation or proton therapy so as not to miss the primary site [18]. However, with an HPV positive cancer, many institutions and authors advocate narrowing the field to the oropharynx only, but this still presents a much larger area than typical compared to a localized tumor [19]. This treatment volume has significant functional implications. Acute side effects include dermatitis, mucositis, odynophagia, dysphagia, and weight loss, while long‐term complications include persistent xerostomia, dysphagia, G‐tube dependence, osteoradionecrosis, thyroid dysfunction, carotid artery rupture and fistula, and nervous system toxicity [14].

Indocyanine green (ICG) is an FDA‐approved near‐infrared fluorescent dye that has been used in various oncologic surgeries, as it has been found to concentrate within various types of tumors [20, 21, 22]. It presents an exciting new modality of directly visualizing occult cancers and detecting the primary site. Previous studies have demonstrated the utility of ICG in conjunction with the TORS, though these studies have focused on its use with known primary sites [23, 24]. Our objective was to determine if ICG could be used in localizing the primary tumor site in a patient with SCCUP. Herein, we present a case of primary localization of an SCCUP in the head and neck using ICG in the setting of TORS.

## 2 Case 1

A 57‐year‐old woman presented with a 5‐month history of a left neck mass. She had previously undergone a biopsy of the mass that revealed HPV+ positive SCCa. CT imaging revealed a single 4 cm Level II cervical lymph node, with tonsillar asymmetry but no obvious primary lesion. PET/CT had similar findings, with no hypermetabolism noted outside of the neck. One month prior, she had undergone a tonsillectomy with bilateral base of tongue biopsies, all of which were negative for SCCa. Her physical exam was unremarkable, although flexible fiberoptic laryngoscopy did reveal some fullness of the lateral base of the tongue.

**FIGURE 1:**
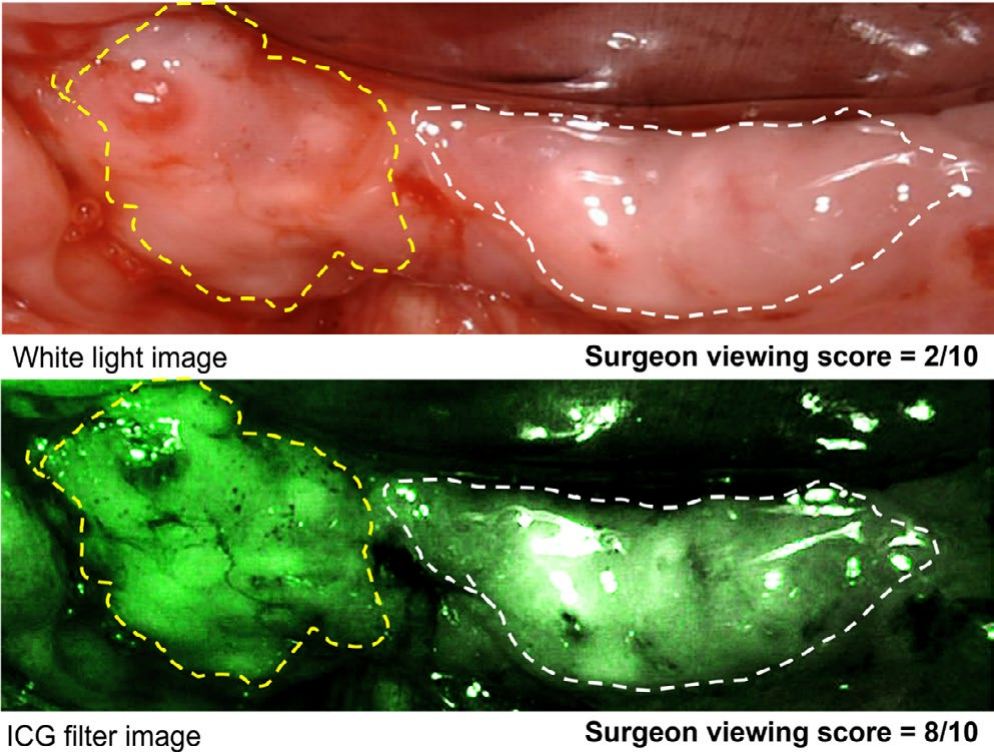
Intraoperative tumor visualization. The yellow dashed line represents the tumor, and the white dashed line represents the normal tissue. The image is of a focused region‐of‐interest (ROI) selected to optimize tumor‐to‐background visualization.

The patient was offered definitive chemoradiation versus TORS with an attempt to identify the primary site and adjuvant therapy as indicated. After a thorough discussion, the decision was made to proceed with operative intervention. The patient was enrolled under a larger prospective, observational cohort study with approval from the Institutional Review Board at the University of Utah (IRB No. 00113447). Six hours before surgery, 25 mg of ICG was administered intravenously. At the time of surgery, the oropharynx was examined under both white light and fluorescent filters. There was a notable increase in fluorescent signal at the left base of the tongue (Figure 1). However, when imaged under the white light, it was difficult to identify the area of concern. As a result, the surgeon gave a surgeon viewing score of 2 out of 10 for white light and 8 out of 10 for ICG filter (viewing score indicates a grading system to visualize the primary tumor with and without ICG fluorescence, adapted from Athanasiadis et al. [25]). Biopsies obtained from the base of the tongue and tonsil were evaluated via frozen intraoperative analysis and did not reveal evidence of carcinoma. Given the notable increase in fluorescence at the left base of the tongue, the decision was made to proceed with a left base of tongue mucosal resection.

**FIGURE 2:**
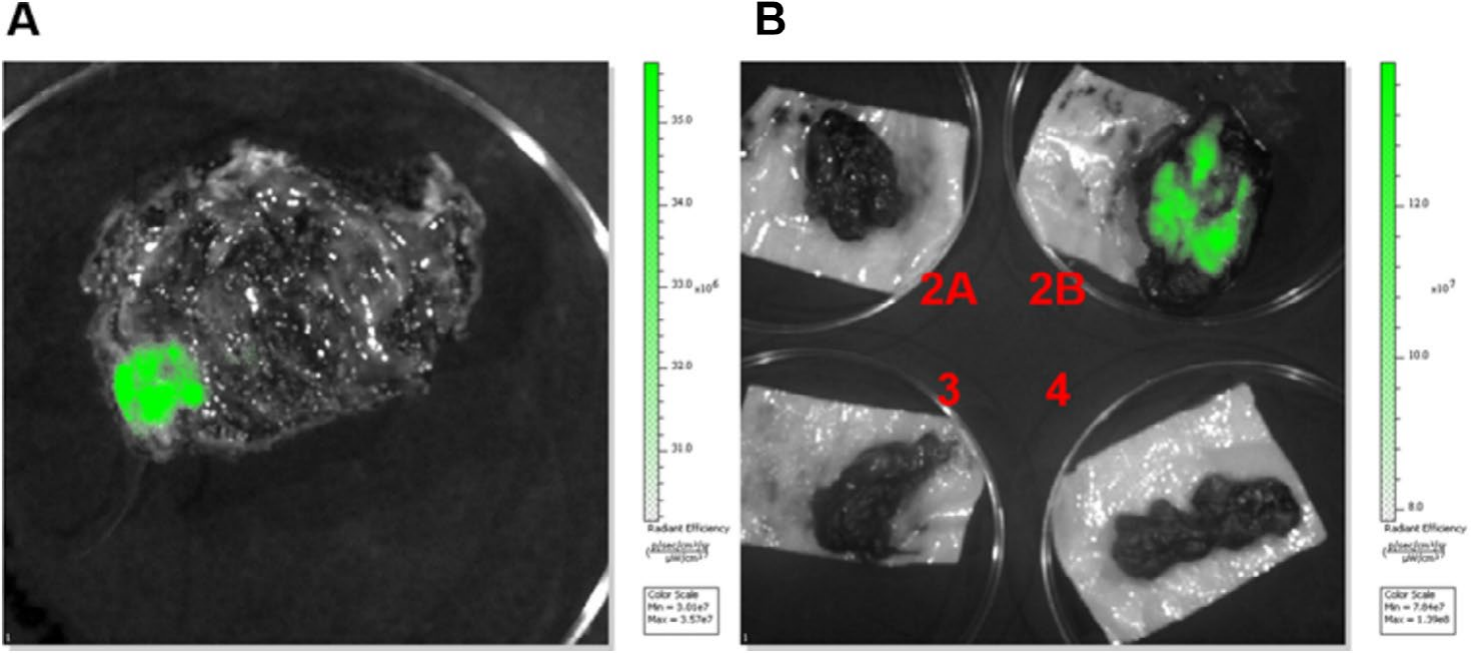
IVIS imaging of primary tumor and lymph nodes after resection (the image shows the whole specimens). Green overlay indicates the presence of (A) primary carcinoma after resection of tissue (IVIS‐derived estimate of lesion was 0.78 ± 0.13 cm) and (B) primary carcinoma after resection of pathological lymph nodes (red indicates the lymph node level).

After resection of the left base of the tongue, the tissue was grossly reevaluated, and no primary site was palpated or noted by the surgeon. The tissues were then taken for in vivo imaging system (IVIS) imaging, which allows quantification of a fluorescent signal and has previously been shown to be effective as an adjunct diagnostic tool to histopathology in identifying carcinoma [23]. IVIS revealed a focus of increased ICG fluorescence in the excised tissue, which was concerning for involvement with cancer, in addition to increased fluorescence along the periphery of the specimen, concerning for a close margin (Figure 2A). Therefore, the decision was made to proceed with a formal TORS base of tongue resection and modified radical neck dissection. On final pathology, the left base of tongue specimen was found to be positive for HPV+ SCCa, and all surgical margins were negative. However, no gross or microscopic tumor dimension was reported in the pathology report. The only histopathologically positive lymph node was the one biopsied at the time of presentation, which was confirmed via IVIS imaging postsurgical resection of lymph nodes (Figure 2B).

Postoperatively, the patient was offered adjuvant radiation therapy (RT) versus observation based on institutional tumor board recommendation and the ECOG 3311 results [17]. The patient elected for observation and has been recurrence‐free for over 3 years now, and reports her swallowing function as normal without any limitations.

## 3 Discussion

This case demonstrates the use of intraoperative ICG fluorescent visualization, allowing for de‐escalation of surgery in the case of a SCCa with unknown primary in the head and neck. Without successful localization and resection of her base of tongue SCCa, this patient would likely not have been a candidate for treatment de‐escalation and instead would have been committed to high‐dose chemoradiation. ICG may offer a helpful tool in augmenting primary tumor identification in cases of SCCUP, allowing for potential adjuvant treatment de‐escalation.

Approximately 5% of patients who present with cervical lymph node metastasis with SCCa are unable to have a primary site identified, despite a thorough diagnostic workup [18]. However, the advent of TORS has allowed for increased identification rates of SCCUP. The role of systemic ICG fluorescence imaging for SCCUP has begun to be evaluated beyond case‐level observations. Karadaghy et al. reported early clinical experience integrating systemic ICG fluorescence into the diagnostic evaluation of unknown primary disease (*n* = 19), with true‐positive localization in 10 cases and reported sensitivity of 100% (with lower specificity), supporting feasibility while underscoring the need for further validation and optimization of interpretation thresholds [26]. One study noted an 18% increase in primary site identification when TORS was added to panendoscopy and PET/CT [27]. Identification of the primary allows for treatment de‐escalation to primary surgical management, and lower radiation volume/dose if adjuvant RT is needed [18]. This is particularly true in HPV+ disease [28]. One study exploring treatment de‐escalation after primary site identification via TORS found 46.1% of patients to have volume reduction of RT, 30.1% to avoid contralateral neck RT, and 7.7% to avoid adjuvant therapy altogether [12]. TORS has also become a core component of many ongoing clinical trials exploring treatment de‐escalation of treatment for SCCUP or oropharyngeal carcinoma [29]. At our institution, de‐escalation of RT dose is often pursued following the findings of ECOG‐3311, a Phase II randomized clinical trial that found similar outcomes with reduced dose and standard‐dose postoperative RT in patients with Stage III–IVa HPV+ oropharyngeal cancer [17].

De‐escalation of adjuvant therapy is key to improving outcomes in SCCUP. Identifying the primary site in head and neck SCCUP can enable de‐intensification of adjuvant therapy, including reduction of radiation treatment volumes, which has been associated with early functional benefits likely driven by smaller radiation fields [30]. Diffuse RT causes widespread toxicity to the upper aerodigestive tract, with both acute and long‐term consequences. Patients who are spared from high‐dose RT may avoid difficult adverse events, including mucositis, xerostomia, weight loss, feeding tube placements, dental disease, secondary cancers, and osteoradionecrosis [31, 32]. The patient in this report benefited from the use of intraoperative ICG fluorescent visualization, as it aided in the identification of the primary site, facilitating adjuvant treatment de‐escalation.

ICG presents an avenue to increase the efficacy of TORS in primary site identification. ICG has been found to preferentially collect within tumors, a quality attributed to the enhanced permeability and retention (EPR) effect, which suggests that macromolecules preferentially accumulate in tumors by extravasating through permeable blood vessels [33, 34, 35]. ICG is known to bind to albumin in the blood, a macromolecule of approximately 7–8 nm in diameter [36]. The ICG‐albumin complex, due to its macromolecular nature, will subsequently extravasate into tumor tissue and provide a contrast. The exact mechanism of the ICG accumulation and the resulting contrast in the current study is unknown and subject to further investigation. In addition, the EPR effect can vary from patient to patient, and hence this phenomenon needs to be evaluated on a case‐by‐case basis. The Da Vinci surgical system, used for TORS, has near‐infrared visualization technology that allows for seamless integration during surgery. Receptor‐targeted optical imaging probes are an important complementary approach to non‐targeted ICG. While ICG relies primarily on passive tumor accumulation mechanisms, several “active” targeting strategies have advanced through preclinical development and early clinical translation in head and neck cancer. In particular, EGFR‐targeted antibody–dye conjugates (including cetuximab‐IRDye800CW and panitumumab‐IRDye800CW) have been evaluated in patients to enable tumor‐specific near‐infrared fluorescence for intraoperative visualization and margin assessment [37, 38, 39, 40]. In addition, targeted small‐molecule probes such as PARP1‐targeted fluorophores have demonstrated clinical feasibility for tumor‐specific fluorescence in the upper aerodigestive tract [41]. In the context of SCCUP, such targeted probes may be particularly valuable by improving tumor‐to‐background contrast for (i) intraoperative localization of an occult primary, and (ii) ex vivo specimen mapping to guide margin‐directed histopathologic processing when the gross lesion is subtle or not apparent under white light [39, 42].

This case demonstrates the utility of intraoperative ICG fluorescent visualization: under normal visualization (“white‐light filter”), there was difficulty identifying the location of the tumor, while the use of the ICG fluorescent filter greatly enhanced contrast between the tumor and healthy tissue. This concept has been previously explored with success in oral cavity and sentinel lymph node biopsies [40, 43]. One study explored the use of near‐infrared during endoscopy of mucosal head and neck lesions after infusion of ICG, with a sensitivity and specificity of 90.5% and 90.9%, respectively [44]. Our group recently demonstrated the utility and viability of the use of ICG fluorescent image guidance in the operative treatment of patients with HPV+ oropharyngeal SCCa [23]. The use of ICG in TORS is feasible and worthy of further investigation.

## 4 Conclusion

This case provides a unique case of localization of SCCUP in the head and neck using ICG in conjunction with TORS, allowing for complete tumor resection and avoidance of adjuvant chemotherapy and radiation. Utilization of ICG fluorescence for intraoperative visualization of tumors can improve primary site localization rates in patients with SCCUP in the head and neck. It is a low‐cost and low‐risk tool. Improving primary site localization in SCCUP allows for treatment de‐escalation and decreased treatment morbidity in these patients.

## Funding

This work was supported by the Huntsman Cancer Institute, University of Utah.

## Ethics Statement

The study was conducted under an approved protocol from the Institutional Review Board at the University of Utah (IRB No. 00113447).

## Consent

Patient was consented to the use of data in publications.

## Conflicts of Interest

The authors declare no conflicts of interest.

## Data Availability

The data that support the findings of this study are available on request from the corresponding author. The data are not publicly available due to privacy or ethical restrictions.
